# Optic Neuritis: A Rare Paraneoplastic Phenomenon of Hodgkin's Lymphoma

**DOI:** 10.7759/cureus.5181

**Published:** 2019-07-20

**Authors:** Yasir Khan, Waqas Khan, Nishanth Thalambedu, Ammar A Ashfaq, Waqas Ullah

**Affiliations:** 1 Internal Medicine, Abington Hospital - Jefferson Health, Abington, USA; 2 Internal Medicine, Abington Hospital-Jefferson Health, Abington, USA

**Keywords:** optic neuritis, hodgkin's lymphoma, paraneoplastic

## Abstract

Hodgkin’s lymphoma (HL) is a hematological disorder that has a high cure rate. It usually presents as asymptomatic lymphadenopathy or a mass on chest radiograph along with constitutional symptoms ("B" symptoms such as fever, night sweats, or unintended weight loss) in less than half the cases. Optic neuritis is a demyelinating condition that is rarely associated with HL. We present a case of HL that presented with optic neuritis as a paraneoplastic syndrome.

## Introduction

Hodgkin’s lymphoma (HL) is an uncommon disorder with a low incidence rate, comprising only 0.5% of all cancers that will be diagnosed in 2019. HL shows two peaks-the first one in the third decade and the second peak after age 50. It is considered a potentially curable disease with a five-year survival rate of almost 87%. HL is classified by the World Health Organization as nodular sclerosing, mixed cellularity, lymphocyte depleted, lymphocyte rich and nodular lymphocyte-predominant. Patients with HL usually present with asymptomatic lymphadenopathy, unexplained weight loss, and drenching night sweats.

Paraneoplastic syndrome is a group of symptoms that occur with increased frequency in cancer patients. These are not caused by metastasis, direct infiltration of the tumor, or known indirect mechanisms such as toxicity, ectopic secretion of hormones, or induced coagulopathies. Paraneoplastic syndrome is thought to be caused by an immune mechanism against the antigens that are normally present in the body.

Optic neuritis is an acute demyelinating condition of the optic nerve. It usually occurs in patients in multiple sclerosis but can present as an isolated neurological finding. It typically presents with a sudden loss of vision, which can vary from a small defect in the field of vision to total loss of light perception followed by improvement over several months [1]. Rarely, it appears as a paraneoplastic phenomenon associated with small cell lung cancer (SCLC). We are presenting a case of a 21-year-old man whose optic neuritis was a paraneoplastic phenomenon of his underlying HL.

## Case presentation

A 21-year-old man with no significant past medical history was seen in his college student health department with complaints of bifrontal headaches and blurred vision in the left eye. He was noted to have 20/50 vision in his left eye. He denied any history of fever, chills or night sweats but did report a weight loss of 6 lbs. over the past month. He was referred to the emergency room (ER), and a magnetic resonance imaging (MRI) of his brain showed abnormal elevated T2 signal involving the left optic nerve along the leftward aspect of the chiasm in the left prechiasmatic and intracanalicular portions of the left optic nerve. He was treated with IV methylprednisolone for three days, followed by oral prednisone taper. Whereas his headache responded to the steroid treatment, his vision remained the same. An MRI of the cervical and thoracic spine was obtained to see the lesions of multiple sclerosis. This MRI showed a normal spinal cord with no findings consistent with multiple sclerosis. However, incidentally, we noted bilateral level five cervical adenopathy measuring approximately 3 cm and mediastinal and right hilar adenopathy also measuring up to 3 cm. A lumbar puncture was also done that showed glucose of 79 mg/dl, protein of 29 mg/dl, and 2 UL white blood cell (WBC). The results of his cytology examination were negative. A computed tomography (CT) of his neck and chest revealed extensive bilateral adenopathy in the lower neck, supraclavicular areas, right paratracheal region, right hilum, and an anterior mediastinal mass measuring 3.1 cm x 5.8 cm.

The patient was discharged from the hospital with an outpatient follow-up for a hematological evaluation. His positron emission tomography (PET) scan showed increased tracer activity associated with lymph nodes in the neck and chest, which was consistent with a clinical history of lymphoma. However, no tracer activity was identified below the diaphragm. He then underwent right cervical lymph node biopsy that showed large nodules encircled by fibrosis with scattered classic and lacunar Reed-Sternberg cells. The patient continued to have blurred vision that had remained the same in the initial outpatient follow-up. He underwent echocardiogram and pulmonary function testing and was started on ABVD (adriamycin, bleomycin, vinblastine, dacarbazine) chemotherapy with a plan for a PET scan after two cycles with a total of four cycles with involved-field radiation therapy (IFRT) depending on the chemotherapy response. Optic neuritis was a paraneoplastic phenomenon. The patient decided to receive chemotherapy at a different hospital and did not follow up with our clinic. Upon calling the patient later, he informed us that he was undergoing chemotherapy, and his symptoms of blurred vision have resolved completely.

## Discussion

Paraneoplastic syndromes are rare with lymphomas. The probability of paraneoplastic syndrome associated with HL is even lower. Demyelinating neuropathies are found more commonly with non-HL [2].

The presence of neurological pathology of unknown origin at the time tumor diagnosed can be entirely coincidental and may be the result of two different but concurrent events. The neurological symptoms are considered definite paraneoplastic syndrome if the patient has well-characterized onconeural antibodies along with the path of the tumor. The paraneoplastic syndrome can also be considered definite in the absence of onconeural antibodies if the neurological symptoms improve after the treatment of the cancer [3].

Paraneoplastic optic neuritis has been reported but usually does not occur as an isolated finding. It mostly happens in association with other neurological syndromes such as retinitis, posterior cerebellar degeneration, or encephalomyelitis [4]. Optic neuritis associated with malignancy has clinical and radiographic features indistinguishable from optic neuritis associated with multiple sclerosis. Because of identical clinical manifestation generated by both conditions, the exact determination of its etiology is critical for proper treatment of the underlying disease. Occasionally, it presents as the initial finding of underlying cancer [5]. Paraneoplastic optic neuritis is most commonly associated with SCLC, but there have been cases reporting it in the setting of non-SCLC, thymoma, renal, and thyroid cancer [6-8]. Many of these patients have antibodies to collapsin-responsive mediator protein-5 [9]. To the best of our knowledge, there have been no reports of optic neuritis occurring as paraneoplastic syndrome with HL.

Patients usually present with loss of vision that may be painless. This finding may be unilateral or bilateral. This may be associated with other neurological symptoms or may occur as an isolated finding. MRI is done to evaluate the cause of optic neuritis and may show enhancing and swollen optic nerve. Treatment of the underlying cancer either through surgical excision or chemotherapy usually improves the symptoms of optic neuritis.

## Conclusions

Although rare, optic neuritis can present as paraneoplastic syndrome associated with HL. Recognition of this paraneoplastic syndrome can lead to early detection and treatment of cancer, which is a crucial initial step of management.
